# DAF-shielded baculovirus-vectored vaccine enhances protection against malaria sporozoite challenge in mice

**DOI:** 10.1186/s12936-017-2039-x

**Published:** 2017-09-29

**Authors:** Mitsuhiro Iyori, Daisuke S. Yamamoto, Miako Sakaguchi, Masanori Mizutani, Sota Ogata, Hidesato Nishiura, Takahiko Tamura, Hiroyuki Matsuoka, Shigeto Yoshida

**Affiliations:** 1 Laboratory of Vaccinology and Applied Immunology, Kanazawa University School of Pharmacy, First Natural Science Building 1A219, Kakuma-machi, Kanazawa, 920-1192 Japan; 2 Division of Medical Zoology, Department of Infection and Immunity, Jichi Medical University, Shimotsuke, Tochigi Japan; 3 Central Laboratory, Institute of Tropical Medicine (NEKKEN), Nagasaki University, Sakamoto, Nagasaki Japan

**Keywords:** *Plasmodium falciparum*, Baculovirus, Malaria vaccine, DAF, Transgenic parasites

## Abstract

**Background:**

Previous studies have shown that the baculovirus-vectored vaccine based on the “baculovirus dual expression system (BDES)” is an effective vaccine delivery platform for malaria. However, a point of weakness remaining for use of this vaccine platform in vivo concerns viral inactivation by serum complement. In an effort to achieve complement resistance, the gene encoding the human decay-accelerating factor (hDAF) was incorporated into the BDES malaria vaccine expressing the *Plasmodium falciparum* circumsporozoite protein (PfCSP).

**Results:**

The newly-developed BDES vaccine, designated BDES-sPfCSP2-Spider, effectively displayed hDAF and PfCSP on the surface of the viral envelope, resulting in complement resistance both in vitro and in vivo. Importantly, upon intramuscular inoculation into mice, the BDES-sPfCSP2-Spider vaccine had a higher protective efficacy (60%) than that of the control vaccine BDES-sPfCSP2-Spier (30%) against challenge with transgenic *Plasmodium berghei* sporozoites expressing PfCSP.

**Conclusion:**

DAF-shielded BDES-vaccines offer great potential for development as a new malaria vaccine platform against the sporozoite challenge.

**Electronic supplementary material:**

The online version of this article (doi:10.1186/s12936-017-2039-x) contains supplementary material, which is available to authorized users.

## Background

Malaria, a global disease caused by *Plasmodium* parasites and transmitted by the bite of infected anopheline mosquitoes, has a profound impact on affected human populations. The World Health Organization (WHO) estimates that there were 212 million new cases and 429,000 deaths attributable to malaria in 2016 [1], wherein the majority of deaths occurred in African children aged below 5 years. Global malaria elimination campaigns have led to decreasing numbers of malaria cases by the use of insecticide-treated bed nets, indoor-residual spraying of insecticides, rapid diagnostic tests and artemisinin-based combination therapies. However, the appearance of drug-resistant parasites and insecticide-resistant mosquitoes continue to hinder elimination of the disease and preclude eradication of it in the foreseeable future. A safe and effective malaria vaccine is potentially a cost-effective intervention. The most advanced malaria vaccine to date, RTS,S, a subunit vaccine consisting of a portion of the major surface protein *Plasmodium falciparum* circumsporozoite protein (PfCSP), partially decreased clinical events over time in Phase III trials, providing short-lived vaccine efficacy depending on the age group and malaria transmission intensity [2, 3]. Recently, a radiation-attenuated *P. falciparum* sporozoite malaria vaccine, the PfSPZ vaccine, was reported to confer sterile protection against homologous/heterologous controlled human malaria infection in malaria-endemic areas [4–7], which is encouraging news for malaria vaccine developers. However, to achieve an 80% vaccine efficacy by the date proposed by the WHO (2025), further improvement and/or alternative vaccine strategies are urgently needed.

A ‘‘baculovirus dual expression system (BDES)’’, which drives malaria antigen expression by a dual promoter consisting of baculovirus-derived polyhedrin and mammal-derived cytomegalovirus (CMV) promoters, has been developed [8–16]. This system can induce strong anti-malaria parasite immunity in animal models, and has been shown to be a malaria vaccine platform that elicits strong efficacy for targeting all parasite life stages, including the pre-erythrocytic stages [8, 9, 14, 15], the blood-stages [10, 11] and the mosquito-stages [12–16]. A study with a rhesus monkey model showed that BDES vaccines are safe and well tolerated with acceptable reactogenicity and systemic toxicity [14]. Thus, additional studies are now being conducted to improve further the protective efficacy of BDES vaccines.

Key to improving the efficacy of BDES vaccines is evidence that baculoviruses are inactivated by the complement system [17]. Several research groups have been successful in protecting baculovirus from complement attack by displaying decay-accelerating factor (DAF) on the surface of the viral envelope, resulting in a higher transduction efficacy in the presence of complement in vitro [18–21]. To date, no DAF-display baculovirus has been reported to exhibit enhanced protective efficacy as a vaccine platform against in vivo animal challenge tests. In the present study, a DAF-display BDES malaria vaccine was constructed to evaluate the protective efficacy of this construct against parasite challenge in mice. The newly-developed BDES malaria “BDES-Spider” vaccine displayed DAF on the viral surface and conferred complement resistance in vitro and stable gene expression in vivo. Importantly, the protective efficacy of the BDES vaccine against transgenic parasite sporozoites expressing *P. falciparum* circumsporozoite protein (PfCSP) was markedly improved by incorporation of DAF. Protective efficacy against the parasites, as well as strong humoral immunity, was observed following immunization of mice with the BDES-Spider vaccine.

## Methods

### Recombinant viral vaccines

The BDES-PfCSP1-gp64 virus is identical to the “CMV-full” vaccine reported previously [14]. BDES-sPfCSP2-Spier and BDES-sPfCSP2-Spider were generated de novo. The baculovirus transfer vectors, pFast-polh-EGFP-Piggy-D-sPfCSP2(R) (pFast-sPfCSP2-Spier), pFast-polh-EGFP-p10-hDAF-G-Piggy-D-sPfCSP2(R) (pFast-sPfCSP2-Spider), pFast-GL3-Spider and pFast-GL3-Spier, were generated as described in the Additional file 1. Recombinant bacmids were generated by Tn7-mediated transposition of the gene cassettes in pFast vectors using the Bac-to-Bac system (Life Technologies, Gaithersburg, MD), according to the manufacturer’s instructions. Amplification and purification of the baculoviruses have been described elsewhere [14, 15].

### Negative-stain transmission electron microscopy

For immunostaining, baculovirus sample solution was adsorbed on each 200-mesh copper grid with carbon-coated plastic film (Nisshin EM, Tokyo, Japan) and then incubated with an anti-DAF (human CD55) mouse monoclonal Ab (Millipore, Temecula, CA) in phosphate-buffered saline. After washing with PBS, each grid was incubated with a 5 nm colloidal gold-conjugated goat anti-mouse IgG Ab (BBI solutions, Cardiff, UK) in PBS. After washing again, each grid was negatively stained with 1% uranyl acetate solution for 10 s. To test the effect of the serum on the baculovirus samples, the baculovirus sample solution was mixed with heat-inactivated or non-heat-inactivated serum and then incubated for 1 h at 37 °C. The morphology of each baculovirus sample was observed on a JEM-1230 (JEOL, Tokyo, Japan) with an 80 kV acceleration voltage, and images with taken using a 2 k × 2 k Veleta CCD camera (Olympus Soft Imaging Solutions, Lakewood, CO).

### Immunoblotting

Baculoviruses were lysed with loading buffer containing 2% 2-mercaptoethanol, boiled for 5 min. The lysates were separated by 8% SDS-PAGE and transferred to polyvinylidene fluoride membranes, and then probed with either of the following: an anti-PfCSP mouse mAb (2A10) or an anti-hDAF mouse mAb (anti-CD55, Merck Millipore, Temecula, CA), together with an anti-VP39 rabbit Ab [14]. Blots probed with the appropriate secondary Abs conjugated to IRDye 680 and IRDye 800 (Rockland Immunochemicals, Gilbertsville, PA) in the same membranes were visualized using an Odyssey infrared imager (LI-COR, Lincoln, NE). The molecular weight predictions were carried out on the ExPASy server, and densitometry analyses were performed using Image Studio Digits (LI-COR).

### Confocal laser scanning microscopy

For live-cell staining, COS-7 cells (10^4^ cells/well) were transduced with purified baculoviruses at multiplicities of infection (MOIs) of 500. After incubation with the viruses for 48 h, the cells were incubated with Alexa Fluor 594-conjugated anti-PfCSP mAb (2A10) and Syto-13 nucleic acid dye (Invitrogen). The eight-well chambers were mounted with a drop of Vectashield containing 4,6ʹ-diamidino-2-phenylindole (DAPI; Vector Laboratories, Burlingame, CA, USA). An LSM710 inverted laser scanning microscope (Carl Zeiss, Tokyo, Japan) with 20 × and 40 × objectives was used for image acquisition. The mean pixel value of PfCSP, in terms of the expression level, was calculated by Image J software (National Institutes of Health, Bethesda, MD, USA).

### Luciferase assays

Baculovirus (10^7^ pfu) in heat-inactivated (56 °C for 30 min) or intact human serum (Sigma-Aldrich, St. Louis, MO) were incubated at 37 °C for 1 h. HepG2 cells (10^4^) were seeded onto a collagen I-coated 96 well-plate. Viruses (10^6^ pfu) were added and incubated. After 1 h, the virus-containing solution was replaced by culture medium (Dulbecco’s modified Eagle’s medium supplemented with 10% heat-inactivated fetal bovine serum). After 24 h, the culture medium was removed and cell extracts were prepared by addition of cell culture lysis reagent (Promega Corporation, Madison, WI), after which they were assayed following the manufacturer’s instructions (Promega).

### In vivo imaging system (IVIS) for visualizing antigen expression by baculovirus vectors

IVIS (Xenogen Co., Alameda, CA) was used as described previously [22]. After immunization with the GL3-Spider or GL3-Spier vector, anesthetized mice were injected peritoneally with 2 mg of D-luciferin firefly (OZ Bioscience, Marseille, France) and placed in the IVIS camera box for 5 min to count luciferin bioluminescence. The accumulated emissions were calculated, and their intensities expressed in a colour heat map.

### Immunizations and PfCSP-Tc/Pb sporozoite challenge via bites by mosquitoes

Balb/c mice were immunized intramuscularly four times at 3-week intervals with 10^8^ pfu of BDES-sPfCSP1-gp64, BDES-sPfCSP2-Spier or BDES-sPfCSP2-Spider. The method used for the sporozoite challenge infections, which involved infected mosquito bites, has been described previously [14]. Briefly, *Anopheles stephensi* mosquitoes were infected with PfCSP-Tc/Pb, which are transgenic *Plasmodium berghei* parasites expressing PfCSP instead of *P. berghei* CSP. The starved infected mosquitoes were allowed to feed on the abdomen of each mouse for 15 min. The midguts and salivary glands of all mosquitoes were dissected. The mosquitoes that had a blood spot in the midgut and sporozoites in the salivary glands were determined to be infected mosquitoes. All animal care and handling procedures were approved by the Animal Care and Use Committee of Kanazawa University (No. 22118-1). All efforts were made to minimize suffering in the animals.

### Enzyme-linked immunosorbent assay (ELISA)

Sera from immunized mice were collected from tail blood samples 3 weeks after the first, second, and/or third immunizations and 2 weeks after last immunization. PfCSP-specific Ab levels were quantified by ELISA. Pre-coated EIA/RIA plates (Corning Inc.; Corning, NY, USA) with 0.4 µg/well of rPfCSP (*Escherichia coli*-produced) were blocked with 1% bovine serum albumin in PBS and incubated with serial dilutions of sera from the immunized and control mice. In regard to anti-baculovirus IgG titers, homogenates of the purified wild-type baculovirus (10^6^ pfu/well) were used to coat the ELISA plates. Titers of the total IgG, IgG1, IgG2a, and IgG2b specific for the above antigens were detected using horseradish peroxidase (HRP)-conjugated anti-mouse IgGs as described previously [10]. Endpoint titers were expressed as the reciprocal of the last dilution that gave an optical density at 414 nm of 0.15 U above the values of the negative controls (< 0.1). All mice used were seronegative prior to immunization.

### Statistical analyses

Statistical differences between the experimental groups were analysed by the methods described in the individual figure legends; comparisons in which the *p* values were < 0.05 were considered statistically significant. Briefly, between-group differences for the luciferase assays and the antibody responses were assessed by the Kruskal–Wallis test with Dunn’s correction for multiple comparisons. For IVIS, luciferase expression in BES-GL3-Spier- and BES-GL3-Spider-immunized Balb/c mice for individual time points were compared using Mann–Whitney test. A two-tailed Fisher’s exact probability test was performed to determine statistical differences in the protective efficacies of the vaccines using SPSS software (version 19, Chicago, IL, USA). All other statistical analyses were performed using Prism version 6 (GraphPad Software Inc., La Jolla, CA, USA).

## Results

### Construction of a baculovirus dual expression system expressing PfCSP and hDAF

**Fig. 1:**
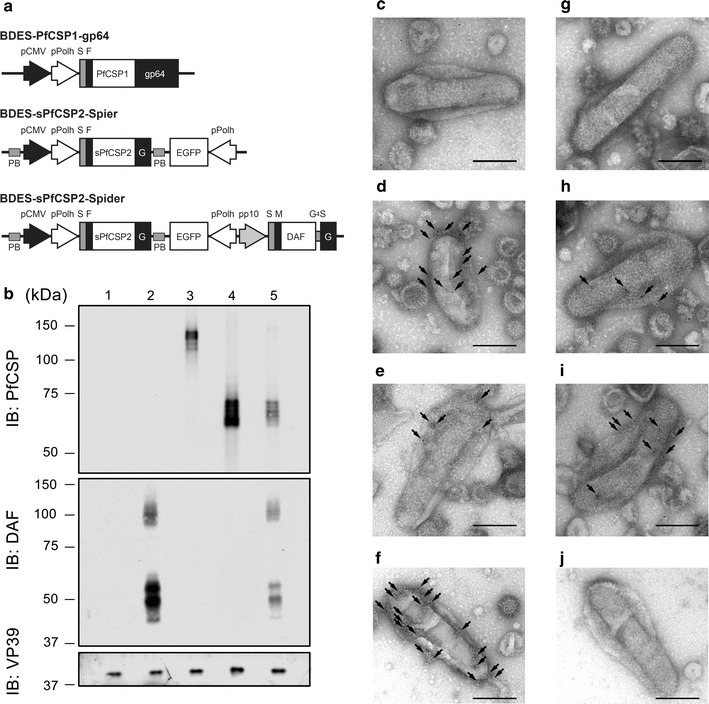
Schematic representation of BDES constructs and the expression of functional proteins. **a** The *pfcsp* genes were cloned from *P. falciparum* (PfCSP^19–373^; PfCSP1) or synthesized with codon-optimization (synthesized PfCSP^19–377^; sPfCSP2). Both *pfcsp* genes were fused to the N-terminus of the *gp64* gene (gp64^21–512^) and the transmembrane region of the *vsv*-*g* gene (VSV-G^421–511^; G). Expression of the PfCSP gene cassette was driven by a dual promoter (pCMV and pPolh). In the “Spider” type of BDES construct, but not in the “Spier” type construct, the human *daf* gene (hDAF) was displayed on virions under the control of the p10 promoter. **b** AcNPV-WT (non-recombinant control), BES-GL3-Spider (hDAF-displayed control), BDES-PfCSP1-gp64, BDES-sPfCSP2-Spier, and BDES-sPfCSP2-Spider (lanes 1–5, respectively) were lysed and subjected to the immunoblots with anti-PfCSP, anti-hDAF, or anti-VP39 Abs. (**c**–**j**) The morphologies of BDES-sPfCSP2-Spider (**c**–**f**) and BDES-sPfCSP2-Spier (**g**–**j**) are shown by transmission electron microscopy. The viral particles were reacted with a nonspecific mouse IgG (**c**, **g**), anti-FLAG mAb (**d**, **h**), anti-PfCSP mAb (**e**, **i**), or anti-DAF mouse mAb (**f**, **j**), and then incubated with a 5 nm colloidal gold-conjugated secondary Ab. Bars, 100 nm; Arrows, colloidal gold signals

It has been reported that the transmembrane region of the G protein of vesicular stomatitis virus (VSV-G TM) enhanced baculovirus-medicated gene transfer in vitro and in vivo [23]. Therefore, the BDES-sPfCSP2-Spier vaccine construct was designed to express codon-optimized PfCSP^19–377^ fused to VSV-G TM to allow PfCSP to be displayed efficiently (Fig. 1a). The BDES-sPfCSP2-Spider vaccine construct was designed to express the human DAF (hDAF) gene in the viral envelope with VSV-G TM, in addition to the gene cassette from the BDES-sPfCSP2-Spier vaccine (Fig. 1a). Both BDES-sPfCSP2-Spier/Spider vaccines include an EGFP sequence under a polyhedrin promoter for use in a reproducible plaque assay system (Fig. 1a).

### Incorporation of PfCSP and hDAF into BDES particles and antigen expression

Immunoblotting showed that BDES-PfCSP1-gp64, BDES-sPfCSP2-Spier and BDES-sPfCSP2-Spider expressed PfCSP in their virions, but a wild-type virus (AcNPV-WT) or a control virus (BES-GL3-Spider, described in the later section) did not (Fig. 1b). The relative molecular mass of BDES-PfCSP1-gp64 was ~ 127 kDa, whereas BDES-sPfCSP2-Spier and BDES-sPfCSP2-Spider were both ~ 60 kDa (Fig. 1b); these values are higher than the predicted molecular weights (103 and 53 kDa, respectively), and possibly result from post-translational modifications or the acidic natures of these proteins (their theoretical isoelectric points are 5.57 and 5.29, respectively). Densitometry showed that the quantity of PfCSP antigen displayed on BDES-sPfCSP2-Spier was over 1.8-fold higher than that of BDES-PfCSP1-gp64 (Fig. 1b). PfCSP expression on BDES-sPfCSP2-Spider was lower than the other vaccines, but hDAF was displayed in the virion (Fig. 1b). The predicted molecular weight of recombinant hDAF is 52 kDa (48 kDa without the signal sequence), and two major bands of around 50 kDa were observed following reaction with the anti-hDAF antibody (Ab) (Fig. 1b).

Transmission electron microscopy showed that nonspecific Ab from a naïve mouse did not react with the BDES particles (Fig. 1c, g), whereas anti-FLAG (Fig. 1d, h) and anti-PfCSP monoclonal Abs (mAbs) (Fig. 1e, i) bound to the BDES particle surfaces, indicating that BDES-sPfCSP2-Spider and BDES-sPfCSP2-Spier both incorporated PfCSP into their viral envelopes. In BDES-sPfCSP2-Spider, hDAF was distributed over the viral surface (Fig. 1f), but BDES-sPfCSP2-Spier did not express hDAF (Fig. 1j). Together, the data support the idea that this vector is able to display both PfCSP and hDAF within a single platform.

**Fig. 2:**
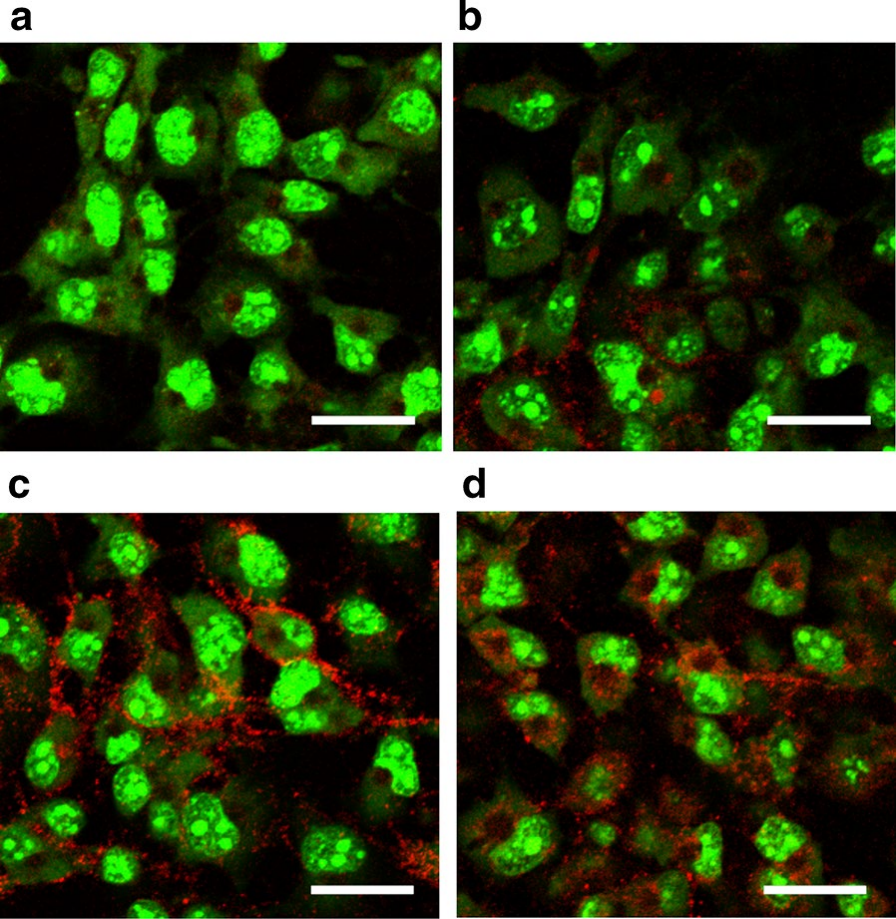
PfCSP gene expression following transduction of the BDES vaccine into mammalian cells. **a**–**d** COS-7 cells were transduced with AcNPV-WT (**a**), BDES-PfCSP1-gp64 (**b**), BDES-sPfCSP2-Spier (**c**) and BDES-sPfCSP2-Spider (**d**) each at a multiplicity of infection of 500. After 48 h incubation, the cells were stained with the 2A10 mAb conjugated to Alexa Fluor 594 (red). Counterstaining of the live cells were conducted by the SYTO-13 nucleic acid dye (green). Bars, 50 µm

Next, the transduction efficacy of the BDES vaccines for mammalian cells was examined. The wild-type virus AcNPV-WT did not induce PfCSP expression (Fig. 2a), while the PfCSP expression levels (mean pixel value) in COS-7 cells that had been transduced with BDES-sPfCSP2-Spier (Fig. 2c) or BDES-sPfCSP2-Spider (Fig. 2d) were two-fold higher than that of BDES-PfCSP1-gp64 (Fig. 2b). The results suggest that BDES-sPfCSP2-Spider/Spier vaccines offer not only antigen-display on the virion but also transduction efficacy of DNA vaccines in mammalian cells.

### Complement resistance of the BDES-sPfCSP2-Spider vaccine

**Fig. 3:**
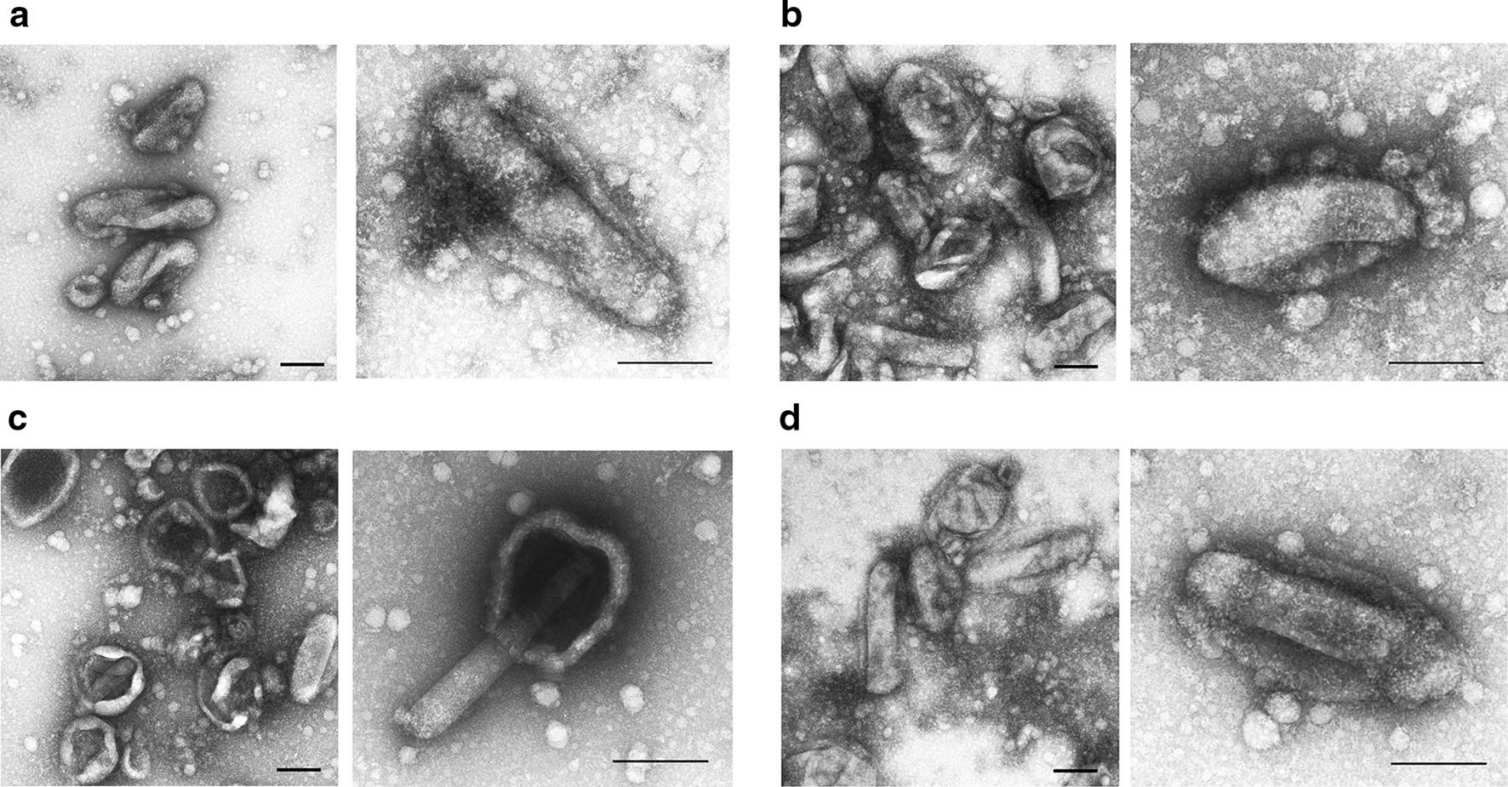
Complement-resistant viral particles following viral incorporation of the BDES-Spider construct expressing human DAF. After the incubation with heat-inactivated or intact human serum, the morphology of each baculovirus was shown by transmission electron microscopy. **a** BDES-sPfCSP2-Spier with heat-inactivated serum, **b** BDES-sPfCSP2-Spider with heat-inactivated serum, **c** BDES-sPfCSP2-Spier with intact serum and, **d** BDES-sPfCSP2-Spider with intact serum. Bars, 100 nm

To investigate the stability of BDES vaccines in the presence of complement, BDES-sPfCSP2-Spider and BDES-sPfCSP2-Spier were examined by electron microscopy. After treatment with heat-inactivated sera, both viruses displayed typical rod-shaped morphologies, which included being envelope-surrounded (Fig. 3a, b). After treatment of intact human sera containing active complement, the BDES-sPfCSP2-Spier particles became aggregated, and the envelopes had fallen off the majority of them (Fig. 3c). In contrast, the BDES-sPfCSP2-Spider particles were morphologically unaffected by complement (Fig. 3d).

**Fig. 4:**
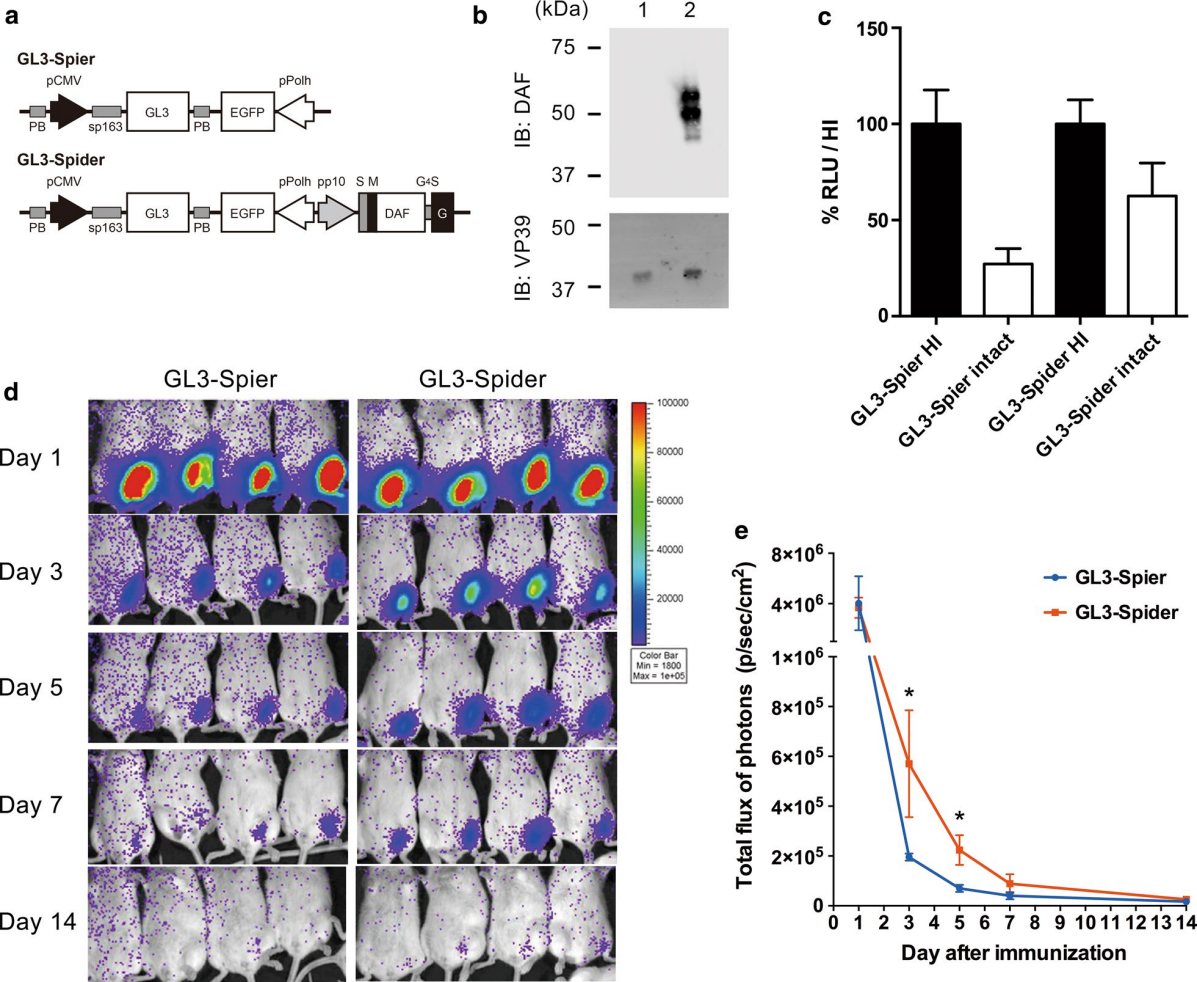
Schematic representation of the luciferase-expressing vaccines and their complement resistance in vitro and in vivo. **a** BES-GL3-Spier and BES-GL3-Spider vectors express firefly luciferase GL3 under the pCMV single promoter, and the BES-GL3-Spider vector contains a gene cassette for hDAF display. PB, PiggyBac transposon sequence; S, gp64 signal sequence; F, FLAG epitope tag; M, myc epitope tag; and EGFP, enhanced green fluorescent protein. **b** BES-GL3-Spier (lane 1) and BES-GL3-Spider (lane 2) were lysed and subjected to immunoblotting with anti-DAF and anti-VP39 Abs. **c** BES-GL3-Spier and BES-GL3-Spider were treated with heat-inactivated or intact serum and then transduced into HepG2 cells. Cell lysates were subjected to luciferase assays. Each relative luciferase unit (RLU) was normalized against each of the values of the heat-inactivated samples (n = 3). Bars and error bars indicate the mean ± SD of the values, respectively. Representative data from three independent studies are shown. HI, heat-inactivated serum; Intact, non-heat-inactivated serum. **d** Luciferase expression in BES-GL3-Spier- and BES-GL3-Spider-immunized Balb/c mice at 1, 3, 5, 7, and 14 days post-immunization (n = 4). The heat map image visible in the mice represents the total flux of photons (p/sec/cm^2^) in that area. **e** The mean total flux of photons is shown. Bars and error bars indicate the mean ± SD of the values, respectively. Comparison between groups was assessed by the Mann–Whitney *U*-test. *p < 0.05, compared with BES-GL3-Spier

To further determine the effect of DAF incorporation into the BDES vaccine in terms of its resistance to complement inactivation, a transduction study was conducted using the following types of vectors based on baculovirus expression system (BES): BES-GL3-Spier, a transiently expressing luciferase vector lacking DAF, and the DAF displaying vector, BES-GL3-Spider (Fig. 4a). Immunoblotting demonstrated that BES-GL3-Spider, but not BES-GL3-Spier, displayed hDAF on their virions (Fig. 4b). The transduction efficacy of the non-DAF-displaying GL3-Spier vector was reduced (20.6%) after treatment with intact sera compared with that of the heat-inactivated sera control (Fig. 4c). In contrast, the GL3-Spider DAF-displaying vector exhibited a substantially higher transduction efficacy than that of the GL3-Spier vector following exposure to intact sera, recovering up to 73.0% of its transduction efficacy observed after treatment with heat-inactivated sera (Fig. 4c). Thus, the complement recovery rate for GL3-Spider was 52.4%. These data indicate that DAF incorporation into BDES particles resulted in the acquisition of complement resistance during transduction into mammalian cells.

### In vivo imaging of antigen expression in mice immunized with BDES vectors

Immunization of mice with BES-GL3-Spier and BES-GL3-Spider resulted in luciferase expression in vivo (Fig. 4d). Luciferase expression levels gradually reduced over time in all mice, but the BES-GL3-Spider-immunized group demonstrated significantly higher luciferase levels than those of the BES-GL3-Spier-immunized group at 3 and 5 days post-vector administration (Fig. 4e). These results indicate that DAF incorporation into the viral virion confers complement resistance in vivo during baculovirus-mediated inflammation.

### Protective immunity acquired by immunization with BDES-sPfCSP2-Spider

**Fig. 5:**
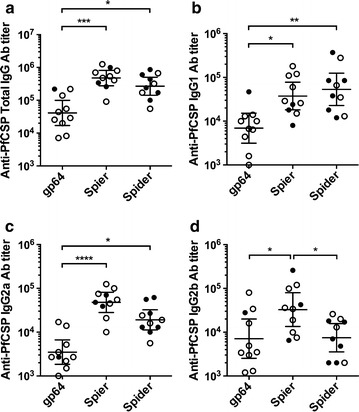
Subclass analysis of anti-PfCSP humoral immune responses. Two weeks after the last immunization, individual serum samples were collected and tested by ELISA for anti-PfCSP total IgG (**a**), IgG1 (**b**), IgG2a (**c**), and IgG2b (**d**). Data from the table is shown (n = 10). Protected individuals are shown as filled circles and unprotected individuals as open circles, and bars and error bars indicate the geometric mean and 95% CI of the values, respectively. Between-group differences were assessed by the Kruskal–Wallis test with Dunn’s correction for multiple comparisons. *p < 0.05; **p < 0.01; ***p < 0.001 and ****p < 0.0001

Next, an experiment was performed to determine whether BDES-sPfCSP2-Spider induced an Ab response against PfCSP in mice and conferred protective immunity against malaria parasites. In agreement with the amount of PfCSP expressed on the BDES particles, the Ab titers [geometric mean and 95% confidence intervals (CI)] after the forth immunization with BDES-sPfCSP2-Spier (476,103; 95% CI 277,053–818,162) and BDES-sPfCSP2-Spider (267,227; 95% CI 141,998–502,896) were significantly higher than that of BDES-PfCSP1-gp64 (41,224; 95% CI 17,019–99,853) (Fig. 5a). An IgG isotype analysis revealed that all BDES vaccines could induce IgG1, IgG2a, and IgG2b (Fig. 5b–d), indicating balanced Th1/Th2 immune responses. The T cell response against PfCSP was investigated by using splenocytes from the immunized mice with H-2K^d^-restricted PfCSP peptide, NYDNAGTNL, but BDES immunization did not induce IFN-γ production from CD8^+^ T cells, as previously described [10].

Two weeks after the last immunization with the BDES vaccines, the mice were challenged by natural feeding of PfCSP-Tc/Pb-infected mosquitoes. After the transmission of the sporozoites via the bites of three to seven infected mosquitoes, the blood-stage malaria was monitored up to the day 14, and then sterile protection was determined by the absence of infection. Significant protective efficacy was observed in the mice immunized with BDES-sPfCSP2-Spier (30%) and BDES-sPfCSP2-Spider (60%), but not BDES-PfCSP1-gp64 (10%), compared with the control mice (Table 1). The Ab levels in mice induced by BDES-sPfCSP2-Spider were equivalent to those of BDES-sPfCSP2-Spier (Fig. 5), but the level of protection against the parasites afforded by BDES-sPfCSP2-Spider was higher than that for BDES-sPfCSP2-Spier.

**Table 1:** The protective efficacy against challenge by the bites of mosquitoes infected with PfCSP-Tc/Pb parasites

Vaccine	Dose	No. of uninfected mice/total no. (%)
PBS	4	0/10 (0)
WT-AcNPV	4	0/10 (0)
BDES-PfCSP1-gp64	4	1/10 (10)
BDES-sPfCSP2-Spier	4	3/10 (30)^a^
BDES-sPfCSP2-Spider	4	6/10 (60)^b^

Mice were screened for PfCSP-Tc/Pb blood-stage infections by microscopic examination of Giemsa-stained thin smears of tail blood after challenge infections. Protection is defined as the complete absence of blood-stage parasitemia on day 14 post-challenge

^a, b^Each group of immunized mice were compared with the nonimmunized group (PBS) to test for statistically significant differences using Fisher’s exact probability test. ^a^ *p* < 0.05; ^b^ *p* < 0.01

## Discussion

The present study examined the potential of BDES as a new malaria vaccine platform. In addition to its high vaccine efficacy, this platform has several advantages, especially from a biological safety perspective including (i) low cytotoxicity, (ii) an inability to replicate in mammalian cells, and (iii) an absence of preexisting antibodies. One of the major obstacles in the in vivo use of baculovirus vectors is the virus inactivation by serum complement [17]. Hence, further improvements in the effectiveness of BDES vaccines will require the surface display of complement regulatory proteins such as to enable it to acquire resistance to attack by the complement system [24]. Here, DAF (from humans) and CSP (from the human parasite *P. falciparum*) were introduced into a BDES vaccine, and its protective efficacy against challenge infections in mice with a transgenic parasite line expressing *P. falciparum* CSP via the natural infection route (mosquito bites) was evaluated. The present study clearly showed that the DAF-shielded baculovirus-vectored vaccine enhances protection against malaria sporozoite challenge infections in mice.

DAF, a GPI anchored protein, is broadly distributed among haematopoietic and non-haematopoietic cells. DAF acts in both pathways either by promoting the decay of C3 convertases in the complement cascade or by catalyzing the permanent inactivation of C3 convertases via factor I-induced proteolytic cleavage [25, 26]. Several lines of evidence have shown that DAF incorporated into AcNPV baculoviral particles is capable of conferring resistance to serum inactivation [18–20], although these studies were limited to in vitro assessments. One of these studies reported that complement resistance in baculovirus displaying DAF had 40% efficacy against human sera when the gp64 envelope glycoprotein was used as a fusion partner [18]. Compared with this previous study [18], the GL3-Spider vaccine displaying DAF fused to VSV-G TM exhibited 52% efficacy. The enhanced complement resistance may result from the higher amount of DAF broadly distributed throughout the viral envelope by VSV-G TM (Fig. 1); this may contribute to the virus-modified immune response, thereby producing effective protection against the parasites.

In the present study, codon-optimization as well as the display platform with VSV-G TM could enhance not only the expression levels of the antigen but also humoral immunity against PfCSP. Compared with other recombinant protein-based malaria vaccines (e.g. RTS,S), the BDES vaccine platform not only displays the relevant antigens on its envelope, with native confirmation [8–16], but also expresses appropriately immunogenic protein upon transduction of mammalian cells. In fact, BDES-sPfCSP2-Spider specifically binds to HepG2 cells through heparan sulfate proteoglycans [8, 27], which is synonymous with binding observed to native PfCSP to the sporozoite surface [28, 29]. Under DAF-shielding, “sporozoite-like” CSP-BDES was able to induce neutralizing Abs and protective immune responses more effectively. Another advantage of BDES as a vaccine platform is its adjuvant-free formulation. BDES, an enveloped double-stranded DNA virus, naturally infects insects and possesses strong adjuvant properties that can activate dendritic cell-mediated innate immunity through MyD88/TLR9-dependent and -independent pathways [30]. Recently, Kaikkonen et al, reported that a DAF-display baculovirus could reduce complement-mediated inflammation injury [19]. For gene therapy applications, it is important to manipulate baculovirus vectors to avoid activation of the innate immune response. In contrast, when used as a vaccine platform, the present study showed that DAF-shielded-BDES still maintained an adjuvant effect capable of activating innate immunity.

## Conclusion

Recently, outbreaks of emerging and re-emerging infectious diseases have become a major threat to human health and global stability. Quick actions must be taken to develop effective vaccines to combat such infectious agents. To this end, the development of a novel vaccine platform is, undoubtedly, now urgent. The present study suggests that the DAF-shielded BDES vaccine platform, which has safety and large-scale manufacturing/bioreactor technologies at the heart of its development, has tremendous potential as a generic “next generation” vaccine candidate for malaria as well as for other infectious pathogens.

## Additional file

**Additional file 1: Figure S1.** Schematic representation of pFast-Spider. **Table S1.** Primers used in this study. Supplementary materials and methods.
