# Supplementary material for: DAF-shielded baculovirus-vectored vaccine enhances protection against malaria sporozoite challenge in mice
Source: Malar J. 2017 Sep 29;16:390. doi: 10.1186/s12936-017-2039-x (PMC5622557; doi:10.1186/s12936-017-2039-x)
Supplement: Supplementary file 1 — Additional file 1: Figure S1. Schematic representation of pFast-Spider. Table S1. Primers used in this study. Supplementary materials and methods. [file 12936_2017_2039_MOESM1_ESM.doc]

**Figure S1**


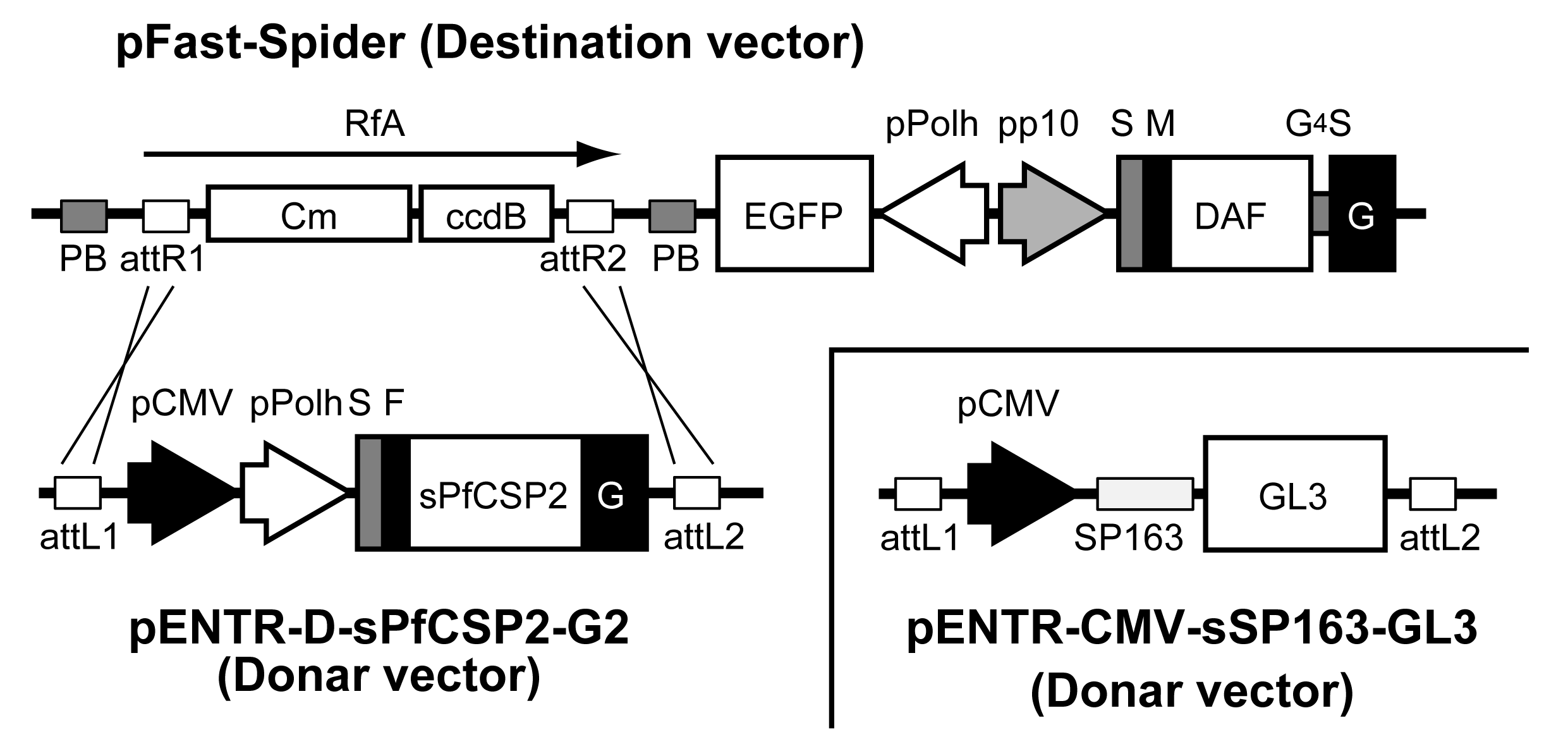


**Fig. S1. Schematic representation of pFast-Spider.** PB, PiggyBac transposon sequence; RfA, reading frame A; Cm, chloramphenicol resistance gene; ccdB, lethal selectable marker gene; EGFP, enhanced green fluorescent protein; S, gp64 signal sequence; M, myc epitope tag; G, transmembrane region of VSV-G; F, FLAG epitope tag; SP163, synthesized QBI SP163 element.

Table S1. Primers used in this study.

| Primer name | Primer sequence |
| --- | --- |
| pBACgus-1 | CTGTAAATCAACAACGCACAG |
| pCMV-F1 | CACCGGCCGGCCTATTAATAGTAATCAATTAC |
| pgp64-F6 | CACCATGCTACTAGTAAATCAGTCACAC |
| pgp64-F7 | CACCGCTAGCCACCATGCTACTAGTAAATCAGTCACACCA |
| phDAF-F2 | CACCGAATTCGACTGTGGCCTTCCCCCAGATGTACCT |
| phDAF-R4 | CCCGGGATCCCCCACCTCCGGAAGTGGTTCCAC |
| pmyc-F1 | GCCTTTGCGGATCTGCAGGAACAAAAACTCATCTCAGAAGAAGATCTGGAATTC |
| pp10-F1 | CACCGGCCGGCCCAATTCCCCGGAGTTAAT |
| pp10-R1 | GGCCGGCCTTTCAGCAAAAAACCCCTCAAGACCCGTTT |
| pPiggy-F1 | CACCCCTAGGTCGCGTCACACAGCTTGGCCACAATGT |
| pPiggy-F2 | CACCTGCAGCTCTAAAATAAGGCGAAAGGCAAATGC |
| pPiggy-R1 | CCTAGGGCGTTGAGATATACGGTATTCACGACAGCA |
| pPiggy-R2 | CTGCAGATCCTCACCAACAAGCTCGTCATCGCTTT |
| pRfA-F1 | CCTGCAGGATCACAAGTTTGTACAAAAAAGCTGAACGA |
| pRfA-R1 | CACCGCATGCATCACCACTTTGTACAAGAAAGCT |
| psPfCSP-R6 | GCATGCACCATAGCACTGGTATTCCTGAAACAGGAATTC |
| pVSV-G-R1 | CTCGAGCCTTCCAAGTCGGTTCATCTCTATGTCTGT |
| pVSV-G-R4 | CACCTCGAGCTAGCCGCGCGCAAGCTTACTTTCCAAGTCGGTTCATCTCTATGTCTGT |
| pVSV-G-R5 | GGTACCTTACTTTCCAAGTCGGTTCATCTCTATGTCTGTATAAAT |
| pVSV-G-F6 | GAGCTCGATTACGAGAATGATATTGAAAAGAAAATTTGTAAGATGGAGAAGTGCAGCTCCGTCCTGCAGGCCCGGGCGTTCGAACATCCTCACATTCAAGACGCTGCT |

**Supplementary materials and methods**

**Plasmid construction**

To construct the pFast-Spider vector, serial DNA cloning was carried out as described below. The schematic representation of this is shown in Figure S1, and the primers used in this study are shown in Table S1.

**Construction of a gene cassette expressing the sPfCSP2-VSV-G TM fusion protein under dual promoter control**. The transmembrane region of glycoprotein G protein cDNA from the vesicular stomatitis Indiana virus was amplified from the pCAP-CO/Full/VSV-G parent vector by PCR with the pVSV-G-F6 forward primer and the pVSV-G-R4 reverse primer, and then ligated to a pENTR-TOPO vector (Life Technologies Inc., Gaithersburg, MD). The resulting vector, pENTR-CSSVLQA-VSV-G2, was digested with *Sac* I and *Xho* I, and the fragment was inserted into a *Sac* I*/Psp* XI cut pTriEx-D-sPfCSP-VSV-G-TM vector (pTriEx-D-sPfCSP2-G2). The DNA sequences of the CMVie and Polh promoters, gp64 signal peptide and FLAG epitope tag were alternatively PCR amplified using pCMV-F1/psPfCSP-R6 primers, and then ligated to a pENTR-TOPO vector to generate pENTR-CMV-Polh. The gene cassette, which included the optimized PfCSP^19-377^ sequence (sPfCSP2), VSV-G TM, and the rabbit β-globin poly (A) signal from pTriEx-D-sPfCSP2-G2, was digested at the flanking *Eco* RI*/Sph* I sites, and then ligated to the same sites of the dual promoter in pENTR-CMV-Polh. The resulting pENTR-D-sPfCSP2-G2 vector was used as a donor vector for Gateway® Cloning.

**Construction of the pENTR-CMV-sSP163-GL3 vector**. The cDNA sequence of the QBI SP163 element of the 5′ untranslated region of the mouse vascular endothelial growth factor gene was synthesized by Genscript (Piscataway, NJ, USA); it included an *Rsr* II site at the 5′ flanking region, a gp64 signal peptide sequence, a FLAG tag epitope sequence and an *Eco* RI site at the 3’ flanking region. The vector was digested with *Rsr* II and *Eco* RI, and then ligated to an *Rsr* II*/Eco* RI-cut pENTR-CMV-Polh vector. The resulting pENTR-CMV-sSP163 vector has an *Nco* I site just after the SP163 sequence. The pcDNA-GL3 vector was constructed previously and is based on pcDNA3 (Life Technologies Inc.) and pGL3-control (Promega). By digesting pcDNA-GL3 with *Nco* I and *Sph* I, the resulting fragment included the firefly luciferase gene (GL3) and SV40 poly (A) signal sequences; this fragment was ligated to *Nco* I*/Sph* I-cut pENTR-CMV-sSP163. This vector, pENTR-CMV-sSP163-GL3, was used as a donor vector for Gateway® Cloning.

**Construction of the RfA cassette with flanking PiggyBac transposon sequences**. pENTR-PiggyL-AvrII-PstI and pENTR-PiggyR-PstI-AvrII vectors were generated by PCR amplification of the pBac3XP3EGFP-pAAPP-mDsRed-2A10scFv-polyA vector with pPiggy-F1/pPiggy-R2 and pPiggy-F2/pPiggy-R1, respectively, followed by ligation to a pENTR-TOPO vector. The pENTR-Piggy-AvrII vector was generated by ligating pENTR-PiggyL-AvrII-PstI and pENTR-PiggyR-PstI-AvrII after digestion with *Pst* I and *Bss*H II. The pENTR-RfA-Sse8387I/Sph I vector was generated by PCR amplification of the pMinos-EGFP-RfA-F vector with pRfA-F1/pRfA-R1 primers, followed by ligation to a pENTR-TOPO vector. The RfA DNA fragment obtained by digestion of the pENTR-RfA-Sse8387I/Sph I vector at the restriction enzyme sites indicated was ligated into *Sph* I*/Pst* I cut pENTR-Piggy-AvrII to generate pENTR-RfA-AvrII.

**hDAF display cassette construction.** To generate a gene cassette expressing the human decay-accelerating factor (hDAF) gene under the control of the p10 promoter, a myc tag-fused antigen displaying pENTR-p10-myc-PfCSP-G-rgt-fsei vector was constructed. To construct the pTriEx-myc-PfCSP-G plasmid, the FLAG tag sequence of the pTriEx-Dual-PfCSP-G vector was replaced by PCR amplification with the pmyc-F1 forward primer, which included the EQKLISEEDL myc tag sequence and *Pst* I*/Eco* RI sites, and the pVSV-G-R1 reverse primer, followed by subcloning into the parent vector via digestion with *Pst* I*/Xma* I. For the addition of the p10 promoter sequence, pTriEx-p10-myc-PfCSP-G was constructed. The open reading frame sequence of pTriEx-myc-PfCSP-G was PCR amplified using the pgp64-F6 forward primer and the pBACgus-1 reverse primer, and then inserted into the pTriEx-3 vector at the *Nco* I (Klenow blunt-ended) and *Xho* I sites. The pENTR-p10-myc-PfCSP-G-fsei vector was constructed from the PCR product using the forward primer pp10-F1 and the reverse primer pp10-R1. This cDNA sequence, which included the p10 promoter, gp64 signal peptide, myc tag, PfCSP, VSV-G transmembrane region, and rabbit globin terminator, was ligated into a pENTR-TOPO vector. To generate the hDAF-expression vector (pENTR-p10-myc-hDAF-G-rgt), the hDAF cDNA sequence was PCR amplified from a DNA sample from human blood with the forward phDAF-F2 primer and the reverse phDAF-R4 primer and then ligated to a pENTR-TOPO vector (pENTR-hDAF). pENTR-hDAF was digested with *Eco* RI and *Xma* I, and then ligated to pENTR-p10-myc-PfCSP-G-rgt-fsei. The resulting pENTR-p10-myc-hDAF-G-TM vector contains the hDAF-display cassette under the control of the p10 promoter.

**pFast-Spider/Spier destination vector construction**. To generate a gene cassette expressing EGFP under the control of the polyhedrin promoter, the previously constructed pcDNA3.1(+)-EGFP vector was digested with *Bam* HI and *Not* I, and then ligated into a *Bam* HI*/Not* I cut pFastBac-Dual vector (Life Technologies Inc.). For restriction enzyme site addition, pENTR-p10-myc-hDAF-G-TM was PCR amplified with the pgp64-F7 forward primer and the pVSV-G-R5 reverse primer, and then ligated to pENTR-TOPO (pENTR-hDAF-VSV-G-NheI/KpnI). The pFastBac-polh-EGFP vector was digested with *Nhe* I and *Kpn* I, and the p10-hDAF-VSV-G cassette was ligated to this vector (pFast-polh-EGFP-p10-hDAF-VSV-G2). Finally, the RfA cassette from the pENTR-RfA-AvrII vector was inserted into pFast-polh-EGFP-p10-hDAF-VSV-G at the *Avr* II/*Pvu* II site. The resulting pFast-polh-EGFP-p10-hDAF-VSV-G-Piggy-RfA(R) vector was used as the destination vector for Gateway® cloning, and is referred to as pFast-“Spider”, where “Spider” is an anagram of the underlined characters in the vector name and the manufacturer’s initial (Shige, Sota and Shuri). The pFast-Spier vector (pFast-polh-EGFP-Piggy-RfA(R)), which was constructed based on the pFast-Spider vector, lacks the hDAF-expression cassette.

**Baculovirus transfer vector generation**. pFast-polh-EGFP-Piggy-D-sPfCSP2(R) (pFast-sPfCSP2-Spier), pFast-polh-EGFP-p10-hDAF-G-Piggy-D-sPfCSP2(R) (pFast-sPfCSP2-Spider), pFast-GL3-Spider and pFast-GL3-Spier, were generated by LR clonase reactions using LR clonase II (Life Technologies Inc.) with the above donor vectors and destination vectors according to the manufacturer’s instructions.
